# Isolation, Molecular Characterization and In Vitro Propagation of an *Anaplasma platys*-Like Bacterium in Tick Cells

**DOI:** 10.3390/pathogens14090901

**Published:** 2025-09-07

**Authors:** Erica Rodrigues de Matos, Priscilla Nunes dos Santos, Erich Peter Zweygarth, Talys Henrique Assumpção Jardim, Huarrisson Azevedo Santos, Matheus Dias Cordeiro, Bruna de Azevedo Baêta, Lesley Bell-Sakyi, Adivaldo Henrique da Fonseca, Claudia Bezerra da Silva

**Affiliations:** 1 Department of Animal Parasitology, Federal Rural University of Rio de Janeiro (UFRRJ), Seropedica 23890-000, RJ, Brazil; 2 Course of Professional Master’s Degree in Diagnosis in Veterinary Medicine, University of Vassouras, Vassouras 27700-000, RJ, Brazil; 3 Department for Comparative Tropical Medicine and Parasitology, Ludwig-Maximilians-Universität (LMU) München, 80802 München, Bavaria, Germany; 4 Department of Biodiversity, Environment and Evolution, Federal University of Ouro Preto, Ouro Preto 35400-000, MG, Brazil; 5 Department of Epidemiology and Public Health, Federal Rural University of Rio de Janeiro, Seropedica 23890-000, RJ, Brazilmathcordeiro@hotmail.com (M.D.C.);; 6 Department of Infection Biology and Microbiomes, Institute of Infection, Veterinary & Ecological Sciences, University of Liverpool, Liverpool L3 5RF, UK

**Keywords:** cattle, Anaplasmataceae, arthropod cell culture, tick cell line, diagnostic

## Abstract

The family Anaplasmataceae comprises etiological agents of infectious diseases of significant importance. This study aimed to achieve the in vitro isolation and propagation of an *Anaplasma* sp. using tick-derived cell lines. The study was realized in Seropédica municipality, Rio de Janeiro, Brazil. Blood smears from a naturally infected bovine revealed cytoplasmic inclusions in blood cells. To isolate and propagate the organism, IDE8 and ISE6 tick cell lines derived from *Ixodes scapularis* were used. Two methods of inoculum preparation were employed: Histopaque^®^ density gradient and platelet-rich plasma separation. Following infection, cells were maintained in L-15B medium without antibiotics at 34 °C, and infection was monitored weekly by Giemsa-stained cytocentrifuge smears. After achieving ≥ 70% infection, bacteria were subcultured and successfully cryopreserved and resuscitated. PCR amplification and sequencing of 16S *rDNA*, 23S *rDNA*, *rpoB*, and *groEL* genes were performed for molecular characterization. Phylogenetic analyses revealed that the isolated strain clustered within the *A*. *platys*-like clade. This study reports the successful in vitro isolation, propagation, and cryopreservation of the ‘*A. platys*-like strain Natal’ bacterium in tick cell lines and provides molecular evidence supporting its phylogenetic classification. These findings contribute to the understanding of genetic variability and host–cell interactions of *Anaplasma* spp., laying the groundwork for future research.

## 1. Introduction

The obligate intracellular *Anaplasma platys*-like bacterium belongs to the family Anaplasmataceae (order Rickettsiales). Its identification has occurred incidentally, exclusively through molecular diagnostic techniques and genetic sequencing, usually during investigations targeting other species of the same genus or to detect possible hemoparasitosis. Although *Anaplasma platys* is classically associated with platelets, particularly in canine hosts, one study reported its presence in neutrophilic granulocytes of camels [1], suggesting a broader cellular tropism that may vary depending on the host species. While *A. platys* is primarily recognized as a canine pathogen, the infection caused by *A*. *platys*-like bacteria in cattle is often subclinical, typically characterized by thrombocytopenia [2]. The presence of this bacterium has been recorded in several species and regions over the years. *Anaplasma platys* was identified in cats in Italy [3]; goats, sheep, and cattle in Tunisia [4]; *Rhipicephalus microplus* ticks in China [5]; *Camelus dromedarius* and also its ticks in Tunisia [1]; buffalo in Thailand [6]; goats in China [7]; cattle in Egypt [8]; cattle in Bolivia [9]; and, more recently, in cattle, goats, and *R. microplus* ticks in Argentina [10]. Although the *A. platys*-like organism is recognized as an obligate intracellular bacterium, its transmission cycle has not yet been fully elucidated, and its pathogenesis remains unknown, including the mechanisms by which it invades and persists within the host. The simultaneous presence of the agent in cattle and its ectoparasites [5,10] suggests that ticks may act as biological vectors. There are several gaps in knowledge, both regarding interaction of the bacterium with specific cells and its clinical effects in different host species.

To date, there are no reports in the scientific literature on the in vitro cultivation of this bacterium. Studies using cell culture methods, as well as alternatives to the use of animals, have shown promise for observing and understanding the interactions between cell lines and infectious agents [11,12]. These advances favor the production of antigens, which can be used in the development of diagnostic tests, such as serological tests, in addition to contributing to the formulation of vaccines [12]. In this context, the development of in vitro culture methods emerges as a promising tool to deepen the biological understanding of the agent [13], allowing studies to be carried out, contributing to advances in diagnostic and therapeutic methods. This study reports establishment of an in vitro culture system for an *Anaplasma* sp. from a naturally infected bovine using tick cell lines and provides molecular and phylogenetic evidence to clarify its evolutionary placement, thereby laying the groundwork for future research on *Anaplasma* diversity and host–cell interactions.

## 2. Materials and Methods

### 2.1. Ethical Approval

All procedures were performed according to the ethical guidelines for the use of animal samples as permitted by the Ethics Committee of the Animal Use of the Federal Rural University of Rio de Janeiro (CEUA/UFRRJ), number 2134171215, with data approval on 29 March 2018.

### 2.2. Strain Origin

*Anaplasma* sp. was isolated from the blood of a calf belonging to the Parasitic Diseases Laboratory, Federal Rural University of Rio de Janeiro, located at Seropedica Municipality, Rio de Janeiro State, Brazil. The animal was an eleven-month-old *Bos taurus* calf, housed in an enclosure and released into a paddock (with a size of 4.5 hectares) three months before the initiation of the study, without any tick control measures applied and with access to water *ad libitum*. Blood smears from this animal were examined and suspected basophilic inclusions were observed within platelets and in neutrophil and monocyte cytoplasm.

The blood samples were collected from the jugular vein in vacutainer tubes containing ethylenediaminetetra-acetic acid anticoagulant (EDTA). They were kept in the fridge at 4 °C until the sample processing.

### 2.3. Tick Cell Lines

IDE8 and ISE6 cells lines (embryonic cells from *Ixodes scapularis* ticks) [14,15], sourced from the Tick Cell Biobank, University of Liverpool, were used to perform the *Anaplasma* sp. isolation. The uninfected cells were grown in sealed T25 flasks at 28 °C in L-15B medium supplemented with 5% fetal bovine serum, 10% tryptose phosphate broth, 0.1% bovine lipoprotein concentrate (MP Biomedicals, Santa Ana, CA, USA), 2 mM L-glutamine, 100 IU/mL penicillin, and 100 µg/mL streptomycin. Their development was evaluated by inverted microscope examination (Nikon Corporation, Tokyo, Japan).

### 2.4. Isolation and Propagation of Anaplasma sp.

Before the isolation procedure, an aliquot of 300 µL was collected from the whole blood samples for subsequent DNA extraction under sterile conditions.

The isolation was performed using two methods: separation by density gradient and platelet-rich plasma isolation. The separation by density gradient was carried out using Histopaque^®^ 1083 (Sigma-Aldrich, St. Louis, MO, USA) as described previously [16]. The layer containing the monocytes was washed twice in PBS (pH 7.4) and the pellet was resuspended in culture medium. The platelet-rich plasma isolation protocol was performed [17] with initial centrifugation at 1600× *g* for 10 min, resulting in the separation of red blood cells and plasma containing platelets and leucocytes. The plasma was pipetted and transferred into another sterile tube, and centrifuged again. At this stage, there was separation consisting of platelet-poor plasma above, with a pellet of platelets at the bottom. The platelets were washed twice in PBS (pH = 7.4) by centrifugation at 2000× *g* for 10 min. Following centrifugation, the platelet pellet was resuspended in culture medium (L-15B). Both inocula were used to infect IDE8 cells.

In both isolation protocols, the medium used was L-15B as described previously for uninfected cells, but without antibiotics and further supplemented with 0.1% NaHCO_3_ and 10 mM HEPES [18], and tick cells were maintained in sealed T12.5 flasks. Twenty-four hours after the isolation, the medium was completely replaced in both flasks.

The flasks were kept at 34 °C and 50% of the medium was replaced twice per week. Infection of the cells was evaluated weekly by preparation of Giemsa-stained cytocentrifuge smears and examination by optical microscopy (Olympus BX41, Olympus Corporation, Tokyo, Japan) at 1000× magnification.

An aliquot of 300 µL was collected from each culture for *Anaplasma* sp. detection and characterization. Subculture was carried out when the infection reached a rate ≥ 70%. The culture was resuspended by pipetting and medium containing the infected cells was transferred in a proportion of 1:6 to a new T12.5 flask previously seeded with uninfected tick cells.

### 2.5. Bacterial Semi-Purification and Transfer to ISE6 Cells

Approximately 1 × 10^7^ IDE8 cells infected with bacteria at a rate > 70% were harvested by pipetting and passing the cell suspension ten times through a bent 22-gauge needle. After disruption the cell suspension was centrifuged at 1000× *g* for 5 min. Cytocentrifuge smears of the supernatant were prepared to assess the residual presence of IDE8 cells. After centrifugation the supernatant containing semi-purified bacteria was added to a flask containing uninfected ISE6 cells. The culture conditions and medium used were the same as previously described for IDE8 cells. The ISE6 cell infection was evaluated as outlined above for IDE8 cells.

### 2.6. Cryopreservation

The infected cells in culture medium were centrifuged at 700× *g* for 10 min. The supernatant was discarded, and the infected cell pellet was resuspended in sucrose–phosphate–glutamate buffer (SPG buffer, MilliporeSigma, Burlington, MA, USA) [19] distributed in cryotubes and frozen at −80 °C in a Mr. Frosty container (Sigma-Aldrich, St. Louis, MO, USA). After a minimum period of 90 min, the cryotubes were stored in liquid nitrogen. For resuscitation, infected cultures were thawed rapidly by immersion of the cryotube in a 37 °C water bath and immediately added to a flask of uninfected cells.

### 2.7. DNA Extraction

DNA from all the samples (one blood and two culture samples) was extracted using the Wizard^®^ genomic DNA purification kit (Promega Corporation, Madison, WI, USA) according to the manufacturer’s recommendations for blood extraction. The DNA extracted from cultures was stored undiluted and diluted in ultrapure water at proportions of 1:10 and 1:100. The DNA concentration and purity were evaluated by NanoDrop^®^ spectrophotometer (Thermo Fisher Scientific, Waltham, MA, USA).

### 2.8. Detection and Molecular Characterization

A PCR was carried out on each sample in triplicate to detect the bacteria in the blood and culture using as target the 16S *rDNA* gene [20], and characterization of the cultured bacteria was performed using as targets the 16S *rDNA* gene [20], the *gltA* gene [21], the *rpoB* gene [22], the 23S *rDNA* gene [23] and the *groEL* gene [22]. All these reactions aim to amplify fragments of genes from the family Anaplasmataceae. The primer sets used for amplification of the target genes and characterization of the bacteria are listed in Table 1.

For PCR amplification, the reaction mixtures (25 µL final volume) contained 1X GoTaq Flexi buffer (Promega Corporation, Madison, WI, USA), 0.8 µM of each primer, 0.2 mM of each dNTP, 1 U of GoTaq Hot Start polymerase (Promega Corporation, Madison, WI, USA), and 1 µL of DNA template. MgCl_2_ concentration varied according to the target gene: 1.5 mM for 16S *rDNA*, 2.5 mM for *gltA* and *groEL*, and 2.0 mM for *rpoB* and 23S *rDNA*. For nested PCR of the 16S *rDNA* gene, the same master mix composition was used in both amplification rounds, with 1 µL of the first-round product as the template for the second reaction.

The reactions were carried out in a Bio-Rad T100 ^TM^ Thermal Cycler under the following conditions: 

16S *rDNA* gene: initial denaturation at 94 °C for 5 min, followed by 39 cycles of denaturation at 94 °C for 1 min, annealing at 50 °C for 2 min, and extension at 72 °C for 1.5 min. The final extension was at 72 °C for 7 min.

*gltA* gene: An initial 2 min denaturation step at 94 °C, followed by 44 cycles of denaturation at 94 °C for 30 s, annealing at 53 °C for 60 s, and extension at 68 °C for 60 s. The final extension was at 68 °C for 3 min.

*rpoB*, 23S *rDNA* and *groEL* genes: Initial denaturation at 95 °C for 15 min, followed by 40 cycles of denaturation at 95 °C for 1 min, annealing at 60 °C for 60 s for the 23S *rDNA* gene, 55 °C for 60 s for the *rpoB* gene, and at 50 °C for 60 s for the *groEL* gene, and extension at 72 °C for 1 min. The final extension was 72 °C for 7 min.

Agarose gels at a concentration of 1.5% (UltraPure™ LMP Agarose, Invitrogen, Thermo Fisher Scientific, Waltham, MA, USA) were prepared to observe the PCR products. The electrophoresis run time was 25 min at 135 V for the *rpoB*, 23S *rDNA* and *groEL* genes and 45 min at 75 V for the 16S *rDNA* gene, using a Mini-Sub Cell GT system (Bio-Rad Laboratories, Hercules, CA, USA). The gels were stained with ethidium bromide and visualized on a UV light transilluminator (L-PIX STi, Loccus Biotechnology, Cotia, SP, Brazil).

### 2.9. Sequencing and Phylogenetic Analysis

DNA purification was carried out using an ExoSAP-IT purification kit (Thermo Fisher Scientific, Waltham, MA, USA). The purified PCR products were sequenced in both directions. Sanger sequencing was performed in an ABI 3730xl DNA Analyzer (Applied Biosystems, Foster City, CA, USA).

The bacterial sequences were aligned and edited using DNA Baser Sequence Assembler, version 5.15 (Heracle BioSoft SRL, Romania). Other sequences were selected to create phylogenetic trees according to their size and similarity to the sequences obtained from the isolated bacteria. 

Phylogenetic reconstructions were performed using a dataset containing *groEL*, *rpoB*, 16S *rDNA* and 23S *rDNA* sequences obtained in this study and a dataset of sequences available from Genbank for each gene. As outgroups, *Wolbachia* sp. (CP050531; AF401090; MN383120; MN123014; MH618383), *Rickettsia* spp. (CP000766; CP003341; AE006914; DQ365810; U12459; L36221), *Neorickettsia* spp. (AF401088; AF401089; CP001431; U24396) and *Ehrlichia* spp. (CP006917; CP007480; CP000107; CP040111) sequences also obtained from the Genbank database were chosen. The sequences were aligned in the MAFFT software, version 7.526 (Kazutaka Katoh, Osaka University, Osaka, Japan) [24] with standard options and then visually inspected. After removing misaligned positions with GBlocks [25] a matrix of cured sequences was obtained. The inference of *Anaplasma* phylogeny was conducted under Maximum Likelihood (ML). The ML implemented in the RaxML program [26] was chosen as the best replacement model in JmodelTest implemented in MEGA7 [27]. Support values of clades were evaluated using the RaxML bootstrap self-convergence criterion [26] with best values of pseudo-replicates.

## 3. Results

### 3.1. Examination of Bovine Blood Smear

**Figure 1:**
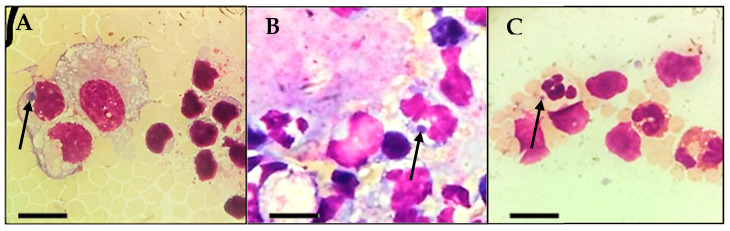
Inclusions found during microscopic observation of blood smears from the calf in the study. (**A**,**B**): Basophilic inclusions in monocytes (arrow). (**C**): Basophilic inclusion in a neutrophil (arrow). Scale bar: 10 µm.

**Figure 2:**
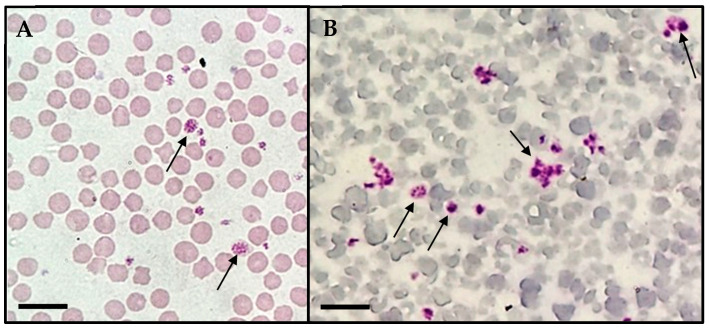
Basophilic inclusions in platelets (arrows) found during light microscopic observation (Olympus BX41^®^, Japan) of blood smears from the calf in the study. (**A**) Macroplatelets with suspected basophilic inclusions. (**B**) Multiple platelets exhibiting dense granules. Scale bar: 10 µm.

During the direct evaluation of blood smears, the sampled calf presented basophilic inclusions in leucocytes (Figure 1A–C) and in platelets (Figure 2). Despite the findings in the blood smears, the animal was not showing clinical signs.

### 3.2. Isolation, Propagation and Cryopreservation of Anaplasma sp. in IDE8 Cells

**Figure 3:**
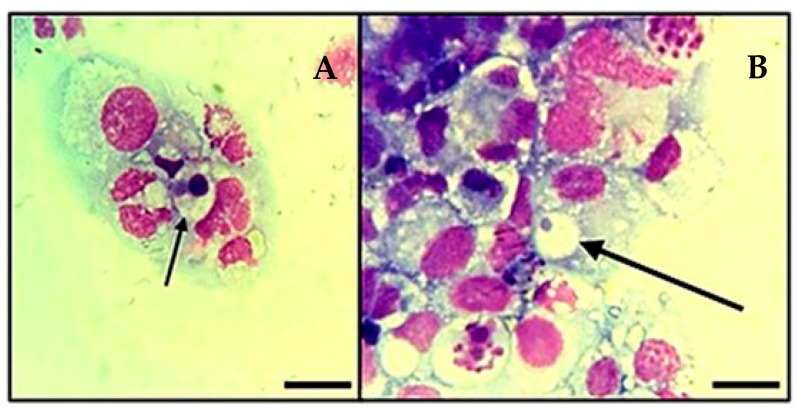
Infection of tick cell line IDE8 with *A. platys*-like strain Natal bacteria. (**A**,**B**): Initial infection of the cells at 7 days post-inoculation. Cytocentrifuge smears of cells in suspension stained with Giemsa; images were taken using a light microscope (Olympus BX41^®^, Japan—×1000), oil immersion; arrows indicate *A. platys*-like morulae; Scale bars = 10 µm.

The cultures showed the first colonies within the cytoplasm of IDE8 cells at day 5 post-isolation with the Histopaque^®^ protocol and day 7 with the platelet protocol (Figure 3). The first subculture was carried out at day 20 for the flask that received white blood cells as the main inoculum and day 28 for the flask inoculated only with platelets. Subsequent subcultures were carried out when the infection reached a rate ≥70% and with an average subculture interval of 14 days.

### 3.3. Bacterial Semi-Purification and Transfer to ISE6 Cells

At passage 16, semi-purified bacteria were transferred from IDE8 cells to a flask containing ISE6 cells. Cytocentrifuge smears of the semi-purified bacterial inoculum, performed to evaluate the presence of IDE8 cells, confirmed the absence of viable tick cells. The growth in the ISE6 cell line was successful and colonies were observed at day 7 after inoculation. The first passage in ISE6 cells was performed 14 days later.

### 3.4. Cryopreservation of the Isolate

**Figure 4:**
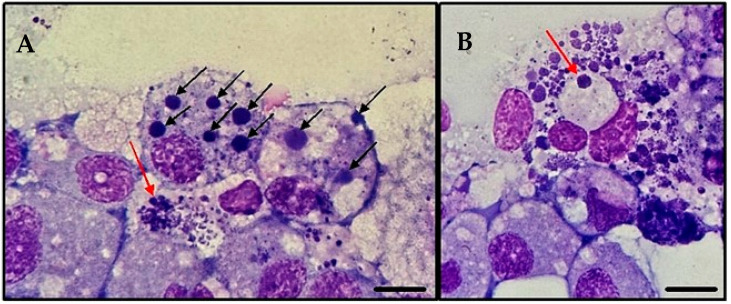
Infection of tick cell line ISE6 with *A. platys*-like strain Natal bacteria, 10 days post-thawing. Cytocentrifuge smears of resuspended cells stained with Giemsa; images taken using an Olympus BX41^®^ microscope; ×1000 oil immersion; (**A**): Large pleomorphic bacteria (red arrow); black arrows indicate *A. platys*-like morulae; (**B**): In addition to the numerous bacteria present in the image, note the formation of a well-defined vacuole (red arrow) containing an inclusion of bacteria.; scale bars = 10 µm.

The cryopreservation was performed on cultures presenting infection rates of ≥70%. The bacteria were first cryopreserved at the first subculture in both IDE8 and ISE6 cells. When resuscitated, the stabilates reinfected the recipient tick cells successfully, and were subcultured 14 days after thawing. The isolate was maintained with continuing growth even after successive freezing and thawing (Figure 4).

### 3.5. Molecular and Phylogenetic Analysis

All PCR assays amplified a DNA fragment, with the exception of the reaction for the *gltA* gene, and consequently there was no molecular characterization of the agent for this target. After amplification of the target genes and trimming the sequences to adjust the alignment, the 16S *rDNA* gene PCR products showed a size of 702 bp, while 23S *rDNA*, *rpoB* and *groEL* showed sizes of 424 bp, 261 bp, and 321 bp, respectively. 

From the analysis of 16S *rDNA*, the sample showed 100% sequence identity (897/897 bp; 100% query cover; 0 gaps; 0.0 E-value) with *Anaplasma* sp. clone Ap-like_WLI (GenBank OQ348131) identified from cattle in South Africa. The 16S *rDNA* gene fragment, which is highly conserved among bacterial species, also showed 100% identity with some sequences previously detected in cattle from South Africa (GenBank: MK814448), impala from South Africa (GenBank: OQ909463) and camels from Saudi Arabia (GenBank: KF843824), notably those related to *Candidatus* Anaplasma camelii (GenBank: KF843824) and *Candidatus* Anaplasma cinensis (GenBank: MF576175). Further analysis of the isolate indicated 97.39% sequence identity (485/498 bp; 100% query cover; 0 gaps; 0.0 E-value) of the 23S *rDNA* gene with *Anaplasma platys* strain S3 (GenBank CP046391) identified from a dog in Panama and 100% identity (458/458 bp; 92% query cover; 0 gaps; 0.0 E-value) with *Anaplasma* sp. clone APC2 (GenBank MN626400) identified from cattle in Egypt.

Analysis of the *groEL* and *rpoB* genes showed 100% sequence identity (531/531 bp; 88% query cover; 0 gaps; 0.0 E-value) with *A. platys* C27 (GenBank LC664076) identified from a bovine in Malawi, and 100% identity (486/486 bp; 94% query cover; 0 gaps; 0.0 E-value) with *Anaplasma* sp. clone APB2 (GenBank MN624139) identified from buffalo in Egypt.

In the phylogenetic trees, the study samples clustered within the same clade as sequences of *A. platys* already found in ruminants from different parts of the world.

**Figure 5:**
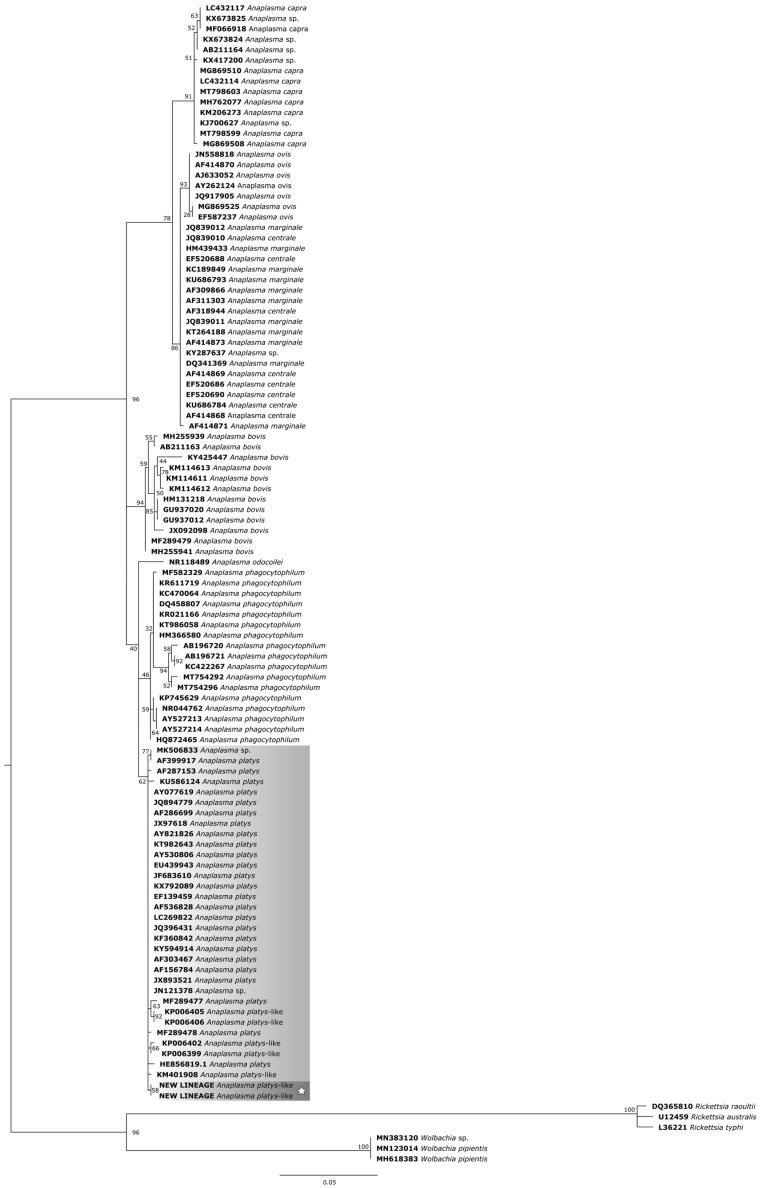
Phylogenetic tree based on the analysis of the 702 bp 16S *rDNA* sequence of *A*. *platys*-like strain Natal bacteria compared to published *Anaplasma* species 16S *rDNA* sequence data. GenBank accession numbers are shown. The tree was constructed using the Maximum likelihood method. *Rickettsia raoultii* (DQ365810), *Rickettsia australis* (U12459), *Rickettsia typhi* (L36221), *Wolbachia* sp. (MN383120), and *Wolbachia pipientis* (MN123014 and MH618383) were used as outgroups. Shaded areas represent sequence clustering, with lighter gray indicating the genetic sequences of *A. platys* and *A. platys*-like strains from previous studies, and darker gray highlighting the sequences analyzed in the present study. Sequences obtained in this study are additionally marked with a star.

For the phylogenetic reconstruction of the 16S *rDNA* gene (Figure 5), the following sequences of *A. platys* and *A. platys*-like bacteria were considered: MK506833 and KX792089 (dogs—Cuba), AF399917, AF287153 and HE856819.1 (dogs—Venezuela), KU586124 (*Anopheles sinensis*—China), AY077619, JX893521 and AF536828 (dogs—Japan), JQ894779, KP006402, KP006405, KP006406 and KP00639 (dogs—Philippines), AF286699 and EF139459 (dogs—Thailand), JX97618, AY821826 and AY530806 (dogs—Spain), KT982643 (dog—India), EU439943 (dog—Italy), JF683610 (dog—Malaysia), LC269822 (dog—Zambia), JQ396431 (dog—Croatia), KF360842 (dog—Panamá), KY594914 (dog—Turkey), AF303467 (dog—France), AF156784 (dog—China), A. sp. JN121378 (*Rhipicephalus sanguineus*—Philippines), MF289477 (cattle—China), MF289478 (cattle—China), KM401908 (dromedary—Tunisia).

**Figure 6:**
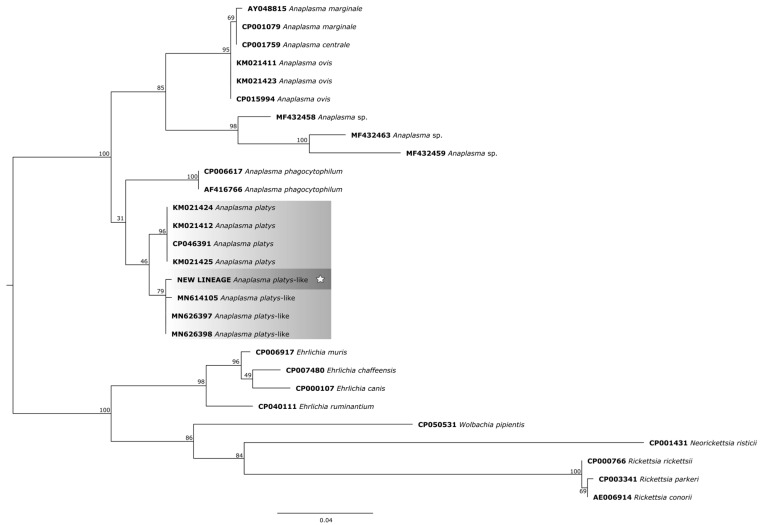
Phylogenetic tree based on the analysis of the 424 bp 23S *rDNA* sequence of *A. platys*-like strain Natal bacteria compared to published *Anaplasma* species 23S *rDNA* sequence data. GenBank accession numbers are shown. The tree was constructed using the Maximum likelihood method. *Ehrlichia muris* (CP006917), *Ehrlichia chaffeensis* (CP007480), *Ehrlichia canis* (CP000107), *Ehrlichia ruminantium* (CP040111), *Rickettsia rickettsii* (CP000766), *Rickettsia parkeri* (CP003341), *Rickettsia conorii* (AE006914), *Neorickettsia risticii* (CP001431), and *Wolbachia pipientis* (CP050531) were used as outgroups. Shaded areas represent sequence clustering, with lighter gray indicating the genetic sequences of *A. platys* and *A. platys*-like strains from previous studies, and darker gray highlighting the sequences analyzed in the present study. Sequences obtained in this study are additionally marked with a star.

For the phylogenetic reconstruction of the 23S *rDNA* gene (Figure 6), the following sequences of *A. platys* were considered: KM021424 and KM021425 (dogs—New Caledonia, France), KM021412 (dog—French Guiana, France), CP046391 (dog—Saint Kitts and Nevis); and for *A. platys*-like bacteria: MN614105 (*Rhipicephalus annulatus*—Egypt), MN626397 (cattle—Egypt), MN626398 (sheep—Egypt).

**Figure 7:**
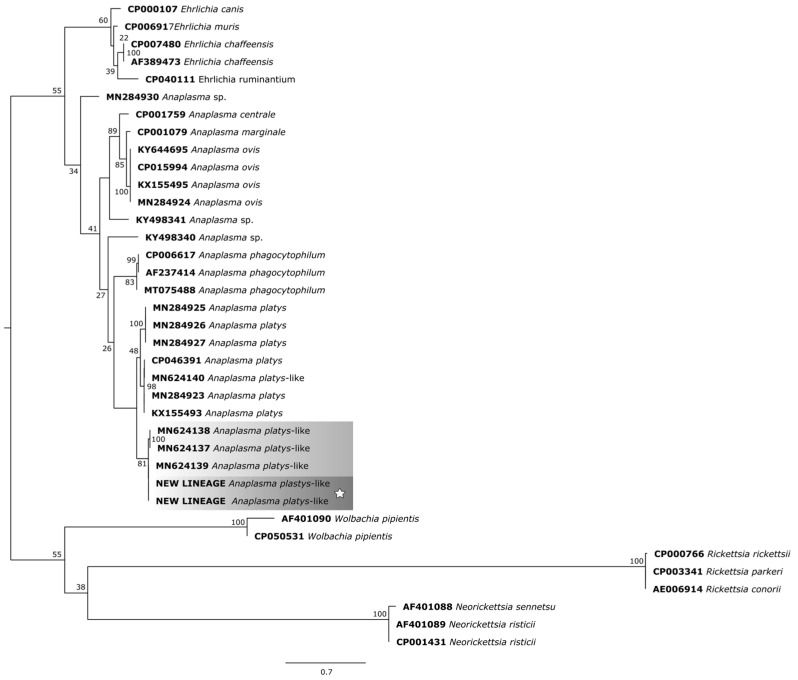
Phylogenetic tree based on the analysis of the 261 bp *rpoB* sequence of *A. platys*-like strain Natal bacteria compared to published *Anaplasma* species *rpoB* sequence data. GenBank accession numbers are shown. The tree was constructed using the Maximum likelihood method. *Rickettsia rickettsii* (CP000766), *Rickettsia parkeri* (CP003341), *Rickettsia conorii* (AE006914), *Neorickettsia sennetsu* (AF401088), *Neorickettsia risticii* (AF401089 and CP001431), and *Wolbachia pipientis* (AF01090 and CP050531) were used as outgroups. Shaded areas represent sequence clustering, with lighter gray indicating the genetic sequences of *A. platys*-like strains from previous studies that are closely related to those analyzed in the present study, which are highlighted in darker gray. Sequences obtained in this study are additionally marked with a star.

For the phylogenetic reconstruction of the *rpoB* gene (Figure 7), the following sequences of *A. platys* and *A. platys*-like bacteria were considered: MN284925 (sheep—Senegal), MN284926 (goat—Senegal), MN284927 (cattle—Senegal); CP046391 (dog—Saint Kitts and Nevis), MN624140 (dog—Egypt), MN284923 (dog—Senegal), KX155493 (dog—France), MN624138 (sheep—Egypt), MN624137 (cattle—Egypt), MN624139 (buffalo—Egypt).

**Figure 8:**
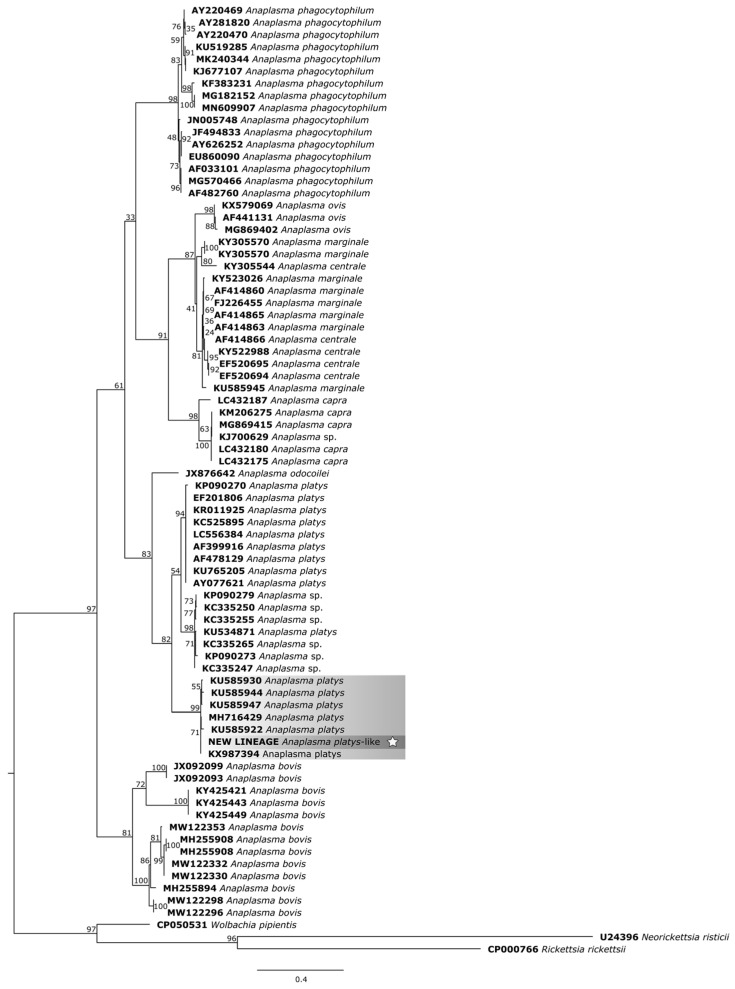
Phylogenetic tree based on the analysis of the 321 bp *groEL* sequence of *A. platys*-like strain Natal bacteria compared to published *Anaplasma* species *groEL* sequence data. GenBank accession numbers are shown. The tree was constructed using the Maximum likelihood method. *Neorickettsia risticii* (U24396), *Rickettsia rickettsii* (CP000766), and *Wolbachia pipientis* (CP050531) were used as outgroups. Shaded areas represent sequence clustering, with lighter gray indicating the genetic sequences of *A. platys* strains from previous studies that are closely related to the sequence analyzed in the present study, which is highlighted in darker gray. Sequences obtained in this study are additionally marked with a star.

For the phylogenetic reconstruction of the *groEL* gene (Figure 8), the sequences of *A. platys* formed a more intimate cluster with our isolate: KU585930 and KU585922 (*Anopheles sinensis*—China), KU585944 and KU585947 (*Armigeres subalbatus*—China), MH716429 and KX987394 (*R. microplus*—China). The other clusters formed based on the *A. platys groEL* gene fragment are mostly derived from samples from dogs, cats, and their associated arthropods.

Based on all analyses performed, the bacterium isolated in tick cell cultures in Brazil was identified as *A. platys*-like. It was designated as ‘*A. platys*-like strain Natal’ with reference to the calf involved in the study, named Natal due to its birth on December 25th, during the Christmas season (in Portuguese, “Natal” means “Christmas”).

## 4. Discussion

This study reports the first successful in vitro isolation and propagation of *Anaplasma platys*-like strain Natal bacteria. While most existing research has focused on molecular diagnostics and phylogenetic characterization, our findings demonstrate the infective potential of this bacterium in tick-derived cell cultures. The establishment of in vitro propagation represents a significant advance, particularly in light of the growing demand for alternative methods that reduce animal use in experimental research. The ability of the bacteria to adapt and replicate in vitro under controlled laboratory conditions offers a valuable platform for future investigations and significantly expands the scope of research in this field.

Tick cell lines have been successfully employed to isolate and propagate members of the family Anaplasmataceae [11,12,15,16,28,29,30,31,32]. Among them, cell lines derived from *I. scapularis* (IDE8 and ISE6) have proven to be valuable in advancing studies on host–pathogen interactions [33], offering a robust in vitro model for exploring the biology and transmission dynamics of these intracellular bacteria. Both tick cell lines utilized in this study also demonstrated remarkable susceptibility to and permissiveness for the isolated agent, enabling efficient cellular interaction and sustained in vitro propagation.

There are many gaps in knowledge about the biology of *A. platys*-like bacteria. One study discussed the evolutionary scenario of the *A. platys*-like strains found in ruminants, their ancestry, and the cellular tropism of the family Anaplasmataceae [34]. Although the original causative agent of infectious canine cyclic thrombocytopenia, *A. platys*, is the only classified rickettsial species known effectively in classical literature to infect platelets, in our study, inclusions related to *A. platys*-like bacteria were found in neutrophils, monocytes and platelets. Similar findings were reported in other studies [1,34] that identified this bacterium in neutrophilic granulocytes. *Anaplasma phagocytophilum* is known to invade neutrophils to cause disease in ruminants [11] and the emerging infection human granulocytic anaplasmosis [35], and *Ehrlichia ruminantium* (previously known as *Cowdria ruminantium*) appears in neutrophils during the onset of heartwater disease in ruminants [36], and for this reason, the molecular tools are useful for the differential diagnosis between agents of the family Anaplasmataceae.

Histopaque^®^ 1083 (Sigma-Aldrich, St. Louis, MO, USA) is a density gradient medium used to separate peripheral blood mononuclear cells (PBMCs) from other blood components, including platelets. While this method effectively isolates PBMCs, residual platelet contamination in the PBMC layer can still occur in methods for density-based mononuclear cell preparation [37]. Although basophilic inclusions suggestive of *A. platys*-like bacterial infection were observed in monocytes and neutrophils in our study, the successful isolation of the bacterium using the platelet-rich plasma (PRP) method, combined with the potential presence of residual platelets in the peripheral blood mononuclear cell (PBMC) separation protocol, suggests a broader cellular tropism. These findings indicate that *A. platys*-like bacteria may not be restricted to a single blood cell lineage but could potentially infect multiple hematopoietic cell types.

Direct detection of *Anaplasma* spp. in blood, tissue, tick, or other vector samples is preferably performed using molecular methods [38]. Approaches involving multiple genes are especially useful in phylogeny and taxonomy studies. The fact that the calf used in this study was asymptomatic reinforces the need for more in-depth investigations into diagnosis, and the clinical and biological aspects relating to infection by this agent. Other direct methods, such as microscopy and in vitro isolation, are mostly used in research, such as experimental studies and transmission assays, but can also contribute to specific diagnostic investigations, surveillance actions, and the identification and characterization of new species of the genus *Anaplasma* [38]. In parallel with DNA extraction from whole blood and culture, blood smears were useful to observe possible inclusions suggestive of intracellular bacteria.

In 2003, tick cell cultures were used to isolate a bacterium known as the “white-tailed deer (WTD) agent” from fawns experimentally inoculated with blood from wild deer [39]. Buffy coat cells were introduced into ISE6 cell cultures, where *Anaplasma* sp. inclusions appeared within 8 days. The isolate, named WTD76, was confirmed by PCR and DNA sequencing to belong to the genus *Anaplasma*, closely related to A. *platys* and *A*. *phagocytophilum*. This study highlighted the relevance of studies with tick cell culture in the characterization of new strains of agents of the family Anaplasmataceae.

Most of the published studies about the investigation and diagnosis of *A. platys*-like bacteria in ruminants were carried out in the Old World, particularly the African continent, using PCRs targeting 16S *rDNA* and *groEL* for detection and molecular characterization of *Anaplasma* spp. in ruminants. Studies conducted at Marromeu Reserve in Mozambique used 16S *rDNA* PCR to reveal *Anaplasma* species infecting African buffalo (*Syncerus caffer*) such as A. *phagocytophilum*, *Anaplasma centrale, Anaplasma marginale*, and *A. platys* [40]. Another study with cattle, also in Mozambique, investigated the occurrence and diversity of *Anaplasma* spp., and high genetic diversity and widespread presence of *Anaplasma* species were observed, including by phylogenetic analysis sequences related to *A*. *platys*, *A*. *phagocytophilum*, *Candidatus* Anaplasma boleense, *A*. *centrale*, *A*. *marginale*, and *A*. *ovis* [41].

In the present study, we chose the target genes already described in the literature to characterize the agent of the family Anaplasmataceae isolated in tick cells. For diagnostic and molecular characterization purposes, this study also employed the primer sets EE1, EE2, EE3, and EE4 [20], which target a partial region of the *A. platys* 16S *rDNA* gene. The sequence obtained from the amplified fragments showed 99–100% identity with multiple *Anaplasma* spp. sequences. When aligned with *Anaplasma platys* strain S3 (GenBank: CP046391), a member of the family Anaplasmataceae typically detected in domestic dogs, our isolate exhibited high identity at the 16S *rDNA* gene (99.89%; 896/897 bp). However, identity values decreased when more variable genes were analyzed, such as 23S *rDNA* (97.39%; 485/498 bp), *groEL* (87.64%; 532/607 bp), and *rpoB* (88.74%; 449/506 bp), supporting previous reports in the literature that suggest the existence of a novel *Anaplasma* sp. genotype infecting cattle [2,6,8,23]. Considering that the 16S *rDNA* gene is a highly conserved target among prokaryotic species, the 100% identity with sequences previously detected in ruminants (including cattle, impala, and camels), as well as with sequences closely related to *Ca.* A. camelii (GenBank: KF843824) and *Ca.* A. cinensis (GenBank: MF576175) [42,43,44,45], highlights the genetic similarity among these DNA sequences. Since the 1990s, the status *Candidatus* has been introduced to bacterial taxonomy, and its use provides a ready solution to the urgent challenge of naming many thousands of newly discovered, but uncultured, species defined by analyses of DNA sequences [46]. Among the genes analyzed in the present study, *rpoB* and 23S *rDNA* had not been previously investigated in the studies that described *Ca.* A. camelii and *Ca.* A. cinensis. On the other hand, analysis of the *groEL* gene revealed low identity (87.78%) between the isolate *A. platys*-like strain Natal and *Ca.* A. camelii, suggesting they represent distinct species. 

The occurrence of *A. platys* in blood samples from cattle was verified in Iraq [2] and these were close to other strains named *A. platys*-China, *A. platys*-Zambia and *A. platys*-Africa, through phylogenetic studies based on the amplification of a partial region of the 16S *rDNA* [20]. *Anaplasma marginale* and *A. platys* were detected and characterized in dairy and indigenous cattle in Vietnam through 16S *rDNA* sequence analysis [47]. Phylogenetic inference placed some of the obtained sequences within the *A. platys* clade. The authors stressed that, given that *A. platys* has been documented as a zoonotic agent [47,48,49], its detection in both cattle and domestic dogs raises significant concerns regarding the potential for zoonotic transmission in Vietnam [48]. Molecular techniques targeting the *groEL* gene were employed to discriminate genetically related strains of *A. platys* in ruminants from northern Tunisia [4]. That study highlighted the importance of molecular tools for epidemiological investigations related to *A. platys*. In a study based on the standardization of molecular techniques, it was evident that the assay based on the 23S *rDNA* gene for the detection of Anaplasmataceae yielded promising results, with 100% specificity [23]. This tool successfully identified several species, including *A. platys* and *A. phagocytophilum*, in cattle from Algeria, where bovine anaplasmosis had not previously been reported. Due to their high genetic variability, the *rpoB* and *groEL* genes are valuable targets for molecular characterization and species differentiation, as observed in the present study. In contrast, the more conserved 16S *rDNA* gene has limited ability to distinguish between closely related species. The 23S *rDNA* gene, although containing conserved regions, has variable regions, making it suitable for detection and species discrimination within the family. *Anaplasma platys*-like and *A. marginale* strains reported as infecting cattle in Egypt showed a considerable degree of genetic divergence. All bovine samples, including cattle and water buffaloes, were tested for *A. marginale* using the *groEL* gene [8].

Data from Thailand indicated, for the first time, that apparently healthy water buffalo were naturally infected by *A. platys*-like bacteria at a relatively high prevalence [6]. The authors concluded that these animals can serve as the reservoir host of anaplasmosis and this is of concern for managing disease control and prevention in ruminants [6]. This study highlights that, despite the high observed prevalence, the absence of clinical signs in the animals suggests that this bacterium may possess low pathogenic potential. In the first molecular study on the diversity and prevalence of *Anaplasma* spp. in privately owned cattle in Kazakhstan [50], one strain showed close similarity to *A. platys* and *Ca.* A. camelii, highlighting the need for further research to clarify the pathogenicity and species range of *Anaplasma* in cattle and ticks in Central Asia. Our phylogenetic analyses revealed that *A. platys*-like bacteria isolated in Brazil are closely related to organisms detected in cattle from South Africa, through molecular techniques targeting the 16S *rDNA* gene, considered a well-conserved target. When we used the 23S *rDNA*, *groEL*, and *rpoB* genes for molecular characterization of this agent, we observed 100% identity with *A. platys*-like sequences found in ruminants from Malawi and Egypt.

Analysis of sequences deposited in genetic databases, combined with a review of international literature, highlights the recurrence of *A. platys*-like bacterial detection in several studies. However, it is observed that these strains are frequently classified directly as *A. platys* (originally described as canine cyclic thrombocytopenia) or assigned to new species, often based on a single molecular marker. This practice can compromise taxonomic accuracy and biological understanding of the agent. It is therefore essential that international nomenclature guidelines be followed and that new studies advance beyond molecular detection, incorporating biological, ecological, pathogenic, and epidemiological aspects. These efforts are essential for the correct understanding and characterization of *A. platys*-like bacteria and for strengthening the scientific literature on the subject.

## 5. Conclusions

This study represents a significant advance in the field of tick-borne pathogen research by achieving the first successful in vitro isolation, propagation, and cryopreservation of *A. platys*-like bacteria from a bovine host. The use of tick cell lines, combined with optimized inoculum preparation techniques, enabled sustained cultivation and reactivation from cryopreserved stocks, demonstrating the biological feasibility of long-term maintenance of this pathogen under controlled laboratory conditions. The findings of this study reinforce the evidence that this bacterium can infect different types of blood cells. These results highlight the need for further investigation to conclusively elucidate the preferred tropism of this pathogen. Molecular and phylogenetic analyses based on multiple gene targets confirmed the taxonomic positioning of the isolate within the *A. platys*-like clade, strengthening the evidence of genetic divergence within the genus. Our findings not only expand the current knowledge of *Anaplasma* genetic diversity and cell tropism, but they also establish a valuable platform for downstream studies involving host–pathogen-vector interactions, pathogenesis mechanisms, and the development of novel diagnostic and therapeutic tools. Ultimately, this work provides a foundation for future research aiming to address the emerging and underestimated risks associated with *A. platys*-like strains in veterinary and public health contexts.

## Figures and Tables

**Table 1:** Primers used in this study.

Target Gene	Primers	Sequence 5′-3′	Size	References
16S *rDNA*	EE-1 EE-2	TCCTGGCTCAGAACGAACGCTGGCGGC AGTCACTGACCCAACCTTAAATGGCTG	1433 bp	[20]
16S *rDNA*	EE-3 EE-4	GTCGAACGGATTATTCTTTATAGCTTGC CCCTTCCGTTAAGAAGGATCTAATCTCC	928 bp	[20]
*gltA*	ANA-CS646F ANA-CS1076R	TGCATGCAGATCATGAAC GAGTAAAARTCAACATTBGG	421 bp	[21]
*rpoB*	Ana-rpoBF Ana-rpoBR	GCTGTTCCTAGGCTYTCTTACGCGA AATCRAGCCAVGAGCCCCTRTAWGG	525 bp	[22]
23S *rDNA*	AnaplatF2 Anagro712R	GCGTAGTCCGATTCTCCAGT CCGCGATCAAACTGCATACC	700 bp	[23]
*groEL*	Ehr-groEL-F Ehr-groEL-R	GTTGAAAARACTGATGGTATGCA ACACGRTCTTTACGYTCYTTAAC	590 bp	[22]

## Data Availability

The original contributions presented in this study are included in the article material. Further inquiries can be directed to the corresponding author.

## Abbreviations

The following abbreviations are used in this manuscript:

CAPES	Coordination for the Improvement of Higher Education Personnel
CEUA	Ethics Committee of the Animal Use
CNPq	National Council for Scientific and Technological Development
DNA	Deoxyribonucleic acid
EDTA	Ethylenediaminetetra-acetic acid anticoagulant
FAPERJ	Carlos Chagas Filho Foundation for Research Support of the State of Rio de Janeiro
FBS	Fetal bovine serum
PBMCs	Peripheral blood mononuclear cells
PBS	Phosphate-Buffered Saline
PCR	Polymerase Chain Reaction
PRP	Platelet-rich plasma
TPB	Tryptose Phosphate Broth
UFRRJ	Federal Rural University of Rio de Janeiro
